# Inhibitory Role of Notch1 in Calcific Aortic Valve Disease

**DOI:** 10.1371/journal.pone.0027743

**Published:** 2011-11-16

**Authors:** Asha Acharya, Chetan P. Hans, Sara N. Koenig, Haley A. Nichols, Cristi L. Galindo, Harold R. Garner, Walter H. Merrill, Robert B. Hinton, Vidu Garg

**Affiliations:** 1 Department of Pediatrics, University of Texas Southwestern Medical Center, Dallas, Texas, United States of America; 2 Department of Molecular Biology, University of Texas Southwestern Medical Center, Dallas, Texas, United States of America; 3 Department of Biochemistry, University of Texas Southwestern Medical Center, Dallas, Texas, United States of America; 4 Center for Cardiovascular and Pulmonary Research and the Heart Center, Nationwide Children's Hospital and Department of Pediatrics, The Ohio State University, Columbus, Ohio United States of America; 5 Virginia Bioinformatics Institute, Virginia Tech Blacksburg, Blacksburg, Virginia, United States of America; 6 Division of Cardiothoracic Surgery, University of Cincinnati, Cincinnati, Ohio, United States of America; 7 Division of Cardiology, Cincinnati Children's Hospital Medical Center, Cincinnati, Ohio, United States of America; Centro Cardiologico Monzino, Italy

## Abstract

Aortic valve calcification is the most common form of valvular heart disease, but the mechanisms of calcific aortic valve disease (CAVD) are unknown. *NOTCH1* mutations are associated with aortic valve malformations and adult-onset calcification in families with inherited disease. The Notch signaling pathway is critical for multiple cell differentiation processes, but its role in the development of CAVD is not well understood. The aim of this study was to investigate the molecular changes that occur with inhibition of Notch signaling in the aortic valve. Notch signaling pathway members are expressed in adult aortic valve cusps, and examination of diseased human aortic valves revealed decreased expression of NOTCH1 in areas of calcium deposition. To identify downstream mediators of Notch1, we examined gene expression changes that occur with chemical inhibition of Notch signaling in rat aortic valve interstitial cells (AVICs). We found significant downregulation of Sox9 along with several cartilage-specific genes that were direct targets of the transcription factor, Sox9. Loss of Sox9 expression has been published to be associated with aortic valve calcification. Utilizing an in vitro porcine aortic valve calcification model system, inhibition of Notch activity resulted in accelerated calcification while stimulation of Notch signaling attenuated the calcific process. Finally, the addition of Sox9 was able to prevent the calcification of porcine AVICs that occurs with Notch inhibition. In conclusion, loss of Notch signaling contributes to aortic valve calcification via a Sox9-dependent mechanism.

## Introduction

Valvular heart disease is responsible for over 20,000 deaths each year in the United States alone, and the aortic valve is the most commonly affected, afflicting an estimated 2.5 percent of adults [1], [2]. Valve calcification leads to stenosis and/or regurgitation that often requires surgical valve replacement. Calcification manifests as clusters of nodules on the arterial aspect of the aortic valve (fibrosa) [3]. The etiology of calcific aortic valve disease (CAVD) is proposed to involve genetic and environmental factors, but the molecular mechanisms underlying the process of aortic valve calcification remain poorly understood [4]–[6].

In vitro model systems using aortic valvular interstitial cells (AVICs) that display osteoblast-like characteristics have provided major insights into the mechanistic basis of calcific valve disease [7]. In addition to the AVICs and overlying endothelial cells, mature adult valves contain a highly diversified and dynamic extracellular matrix (ECM) that exhibits many structural and regulatory characteristics of connective tissues, such as those observed in developing cartilage, tendon and bone [8]. Several collagens and proteoglycans characteristic of cartilage are expressed in developing and mature valves including type II and type IX collagen, cartilage-link protein, and aggrecan and contribute to maintaining the structural integrity of the valve tissue. Supporting the importance of ECM proteins in valve function, loss of ECM organization has been shown to result in valvular maldevelopment and disease [9], [10]. A key transcriptional regulator implicated in this process is Sox9, which is expressed in the developing endocardial cushions and mature valves and when deleted in mice results in valve malformation and calcification [11]–[13]. However, the molecular pathways that regulate Sox9 in valve calcification remain unknown.

Mutations in the transmembrane receptor *NOTCH1* were previously reported to be associated with bicuspid aortic valve and CAVD in human families [14]. These findings not only supported a central role for Notch signaling in valve formation, but also strongly suggested a necessary role for this signaling pathway in the maintenance of normal valve function in adults. The evolutionarily conserved Notch family of receptors regulates a broad spectrum of cell fate decisions and developmental processes during both embryonic and postnatal life [15]. In mammals, activation of Notch receptors (Notch1-4) by their ligands (Jagged-1 and 2 and Delta-like-1, 3 and 4) results in two successive proteolytic cleavages, leading to the release of Notch intracellular domain (NICD) and its nuclear translocation. In the nucleus, NICD functions to initiate transcription of target genes, which include the HEY (Hes-related with YPRW motif) family of transcriptional repressors that have been implicated in early valve development [16]. However, the role of Notch signaling in aortic valve maintenance and CAVD is poorly understood.

Here, we show that diseased human aortic valves have decreased expression of the constitutively active cleaved form of Notch1 in regions of calcification. Using an in vitro primary aortic valve cell culture system, we demonstrate that inhibition of Notch signaling promotes calcification and results in changes in the expression of ECM genes, which are known direct transcriptional targets of Sox9. We find that Notch1 regulates the expression of Sox9 in aortic valve cells and has the ability to activate a Sox9-dependent reporter in vitro. Lastly, we show that overexpression of Sox9 markedly attenuates the calcification that occurs with Notch inhibition suggesting that Notch regulates calcification of aortic valve cells in a molecular pathway mediated by Sox9.

## Methods

### Ethics Statement

Research was approved by the Institutional Animal Care and Use Committee at University of Texas Southwestern Medical Center (Protocol No. 0754-06-03-1) and Research Institute at Nationwide Children's Hospital (Protocol No. AR10-00026) and conforms to the Guide for the Care and Use of Laboratory Animals.

Human valve studies were approved by the Institutional Review Board at Cincinnati Children's Hospital Medical Center and conform to the principles outlined in the Declaration of Helsinki. Written consent was obtained from all participants involved in this study.

### Aortic Valve Interstitial Cell Culture

Rat aortic valve cusps were harvested from adult Sprague-Dawley rats and AVICs were cultured from explants based on published protocols [7]. Briefly, valve leaflets were subjected to collagenase digestion and gently scraped to expose the subendothelial layer. The leaflets were then cut into microscopic pieces (1–2 mm^2^) and cultured in standard Medium-199 supplemented with 15% FBS, 2 mmol/L glutamine and 100 U/ml penicillin/streptomycin. Upon reaching 80% confluency, AVICs were passaged using trypsin-EDTA. AVICs between passage 3 and 8 were used for experiments. Similar protocol was followed for porcine AVIC culture using valve cusps harvested from 3-week old piglets. For rats, euthanasia was performed using inhaled carbon dioxide while pigs were euthanized with a commercially available intravenous mixture of pentobarbital sodium and phenytoin sodium (Euthasol, Virbac, USA). Notch signaling was inhibited using a γ-secretase inhibitor (Sigma Cat. # S2188 or D5942) diluted in DMSO to a concentration of 10 µM, and calcification of rat AVICs was induced by growth in osteogenic media which involved supplementation of standard media with 50 µg/ml ascorbate-2-phosphate, 10 nM dexamethasone and 10 µM β-glycerol phosphate [17]. Culture media was changed every 48–72 hours and cells were passaged at each time point of harvest. Overexpression of Notch targets (Hey1 and Hey2) and Sox9 was achieved using Amaxa Nucleofector technology (Amaxa Inc., USA). Briefly, 0.5–1×10^6^ cells were nucleofected with 5 µg of plasmid DNA using program T16 and incubated until harvesting for described experiments.

### In situ Hybridization

^35^S-labeled antisense riboprobes were synthesized with T7 RNA polymerase (MAXIScript, Ambion) for mouse Notch1, Hey1 and Hey2 as previously described [14]. Radioactive-section *in situ* hybridization was performed on paraffin-embedded sections of hearts from 12-week old albino mice of ICR-CD1 strain following transcardial perfusion as previously described [18]. Non-pigmented albino mice were used to avoid non-specific signals resulting from the presence of pigmented melanocytes that inhabit the valvuloseptal apparatus from embryonic to adult life [19].

### Human Aortic Valve Tissue

Human specimens were obtained from non-syndromic adult patients at University Hospital (Cincinnati, Ohio) who had calcific aortic valve disease and were undergoing aortic valve replacement (mean age  =  64, range 51–82 years), and from age-matched patients at the time of autopsy, who died of non-cardiac causes. Patients with a history of infective endocarditis or rheumatic heart disease were excluded. Tissue was processed and analyzed as previously described [9]. For immunohistochemistry, serial sections (5 µm) from human aortic valves (n = 4 diseased and n = 3 control) were obtained. Antigen retrieval was performed with 0.01M Citrate buffer pH 6.0 for 30 min in a pressure cooker. Blocking was performed with 3% hydrogen peroxide for 15 min, and 5% normal goat serum in PBS Tween20 (0.05% PBST) to reduce non-specific binding. Sections were then incubated with the primary antibody specific to the Notch1 intracellular domain (1∶500 dilution) (Abcam, Cambridge, MA) or Type II Collagen, alpha 1 (COL2A1) (Abcam, Cambridge, MA) overnight at 4°C, washed with PBST, sequentially incubated with biotinylated secondary antibody and avidin-biotin complex (Vector Lab Burlingame, CA), and developed with 3,3′-diaminobenzidine (DAB Peroxidase Substrate Kit, Vector Lab Burlingame, CA). Sections were counter-stained with haematoxylin.

### Gene Expression

For microarray studies, data were RMA normalized and pairwise comparisons (of averaged signal values) and Student's t test with Benjamin and Hoschberg adjustment were performed using GeneSifter (VizX Labs, Seattle, WA). Genes with an average fold-change ≥ 1.5 and an adjusted p value ≤ 0.05 were considered significantly differentially expressed. Gene functions were obtained from Ingenuity Pathway Analysis (Ingenuity® Systems, www.ingenuity.com) supplemented with information from the NCBI and Stanford SOURCE search databases. The microarray data is available on Gene Expression Omnibus (GEO) (Accession # GSE31668) and the microarray data is MIAME compliant. Differential expression of a subset of genes was confirmed by qRT-PCR using RNA isolated from untreated and DAPT treated rat AVICs with gene-specific primers in SYBR Green 1 reactions using Bio-Rad iQ5 Multicolor Real Time PCR machine (Bio-Rad Laboratories). Specific primer sequences are available on request. Fold change in expression between DMSO and DAPT treated samples was determined relative to 18S rRNA, n = 3 per group.

In COS7 cells, Sox9 mRNA levels were measured 48 hours after transient transfection with or without pcDNA-NICD by qRT-PCR. For porcine aortic valves, gene expression studies were performed using Applied Biosystems 7500 Fast Real-Time PCR System. AVICs were cultured up to a four-week time course with either DMSO or 10 µM DAPT in M199 media containing 0.5% FCS. Media was changed every other day. At stipulated timepoints, cells were harvested, washed with cold PBS, and RNA was extracted and analyzed for expression of Notch1, Runx2, alkaline phosphatase and Sox9 (primer sequences are available upon request). Time course experiments were performed in triplicate and qRT-PCR was performed in triplicate and fold change determined by standardization to 18S rRNA and results were normalized to Week 1 levels. Representative qRT-PCR results are shown.

Protein expression was analyzed by Western blotting. Briefly, AVICs were rinsed with cold PBS, proteins extracted with RIPA lysis buffer (Thermo Scientific) and solubilized in 1X SDS Laemmli sample buffer (Bio-Rad Laboratories). Whole cell extracts from porcine and rat AVICs (∼10–15 µg) were blotted on PVDF membranes and blocked with 5% non-fat dried milk in PBS containing 0.5% Tween 20. Immunoblots were probed overnight at 4°C with the following antibodies: Osteopontin (Abcam, 1∶1000), Osteonectin (BioGenex, undiluted), Notch1 intracellular domain (Cell Signaling, 1∶1000), Sox9 (Abcam, 1∶1000), Runx2 (1∶1000), GAPDH (Santa Cruz, 1∶200), and α-tubulin (Sigma, 1∶2000). Immunohistochemistry of human aortic valves was performed using antibody specific for Notch1 intracellular domain (1∶500 dilution) and counter-stained with haematoxylin (Abcam, Cambridge, MA).

### Calcium Staining of AVICs

AVICs were harvested and cultured as described above. After transfection with Sox9 plasmid as detailed above, AVICs were grown on glass chamber slides for six days (Lab-Tek, Rochester, NY). Cells were washed with cold PBS and fixed with 4% paraformaldehyde in PBS for 45 minutes at 4°C. For von Kossa staining, the cells were washed with distilled water and exposed to 5% aqueous AgNO3 and strong light for 60 minutes at room temperature. The cells were then exposed to 2.5% sodium thiosulfate for 5 minutes (black = positive staining) [20]. For Alizarin red staining, the cells were washed in distilled water and then exposed to freshly prepared 2% Alizarin red S (pH to 4.1∼4.3, Sigma) for 5 minutes (red/orange = positive staining) [20]. Digital images were taken on a Zeiss AxioImager upright microscope with brightfield. Calcification of AVICs was assessed using standard protocols for Alizarin red and von Kossa staining. Quantification was performed using Image Pro Plus software.

### Luciferase Reporter Assays

COS7 cells were transiently transfected using Fugene 6 (Roche) with 5-10ng human Sox9 and 10-100ng constitutively active mouse NICD expression plasmids along with 200 ng of the Col2a luciferase reporter plasmid with or without the 4X48 enhancer and 100 ng of β-galactosidase expression plasmid [21], [22]. Luciferase activity was measured 48 hours after transient transfection as previously described [14]. Relative luciferase activities were calculated after normalization for transfection efficiency using β-galactosidase. Three independent experiments were performed in triplicate and means and standard deviations are shown. Statistical comparisons were performed using Student's t-test. P value <0.05 was considered significant. To analyze direct activation of mouse Sox9 promoter by NICD, the endogenous murine Sox9 core promoter (-272 to +1bp) was cloned into pGL3basic as a XhoI-HindIII fragment. DNA fragments encompassing the genomic sequence 5kb upstream of the transcription start site (tss, position +1bp) were then cloned into the core promoter construct as MluI-XhoI fragments. Fold activation by upstream sequences in absence and presence of NICD (0, 100 and 300ng) was determined relative to the core promoter activity.

### Statistics

Statistical comparisons were performed using Student's t-test, and a p value <0.05 was considered significant.

## Results

### Notch1 and Hey Expression in the Adult Mouse Aortic Valve

**Figure 1:**
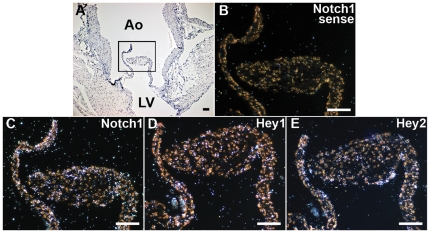
Expression of Notch1, Hey1 and Hey2 mRNAs in adult murine aortic valves by radioactive in situ hybridization. (A) Bright field image of transverse section of 12-week old albino mouse heart. Boxed area is shown in high magnification for (B) Notch1 sense; (C) Notch1 antisense; (D) Hey1 antisense; and (E) Hey2 antisense probes. Ao, aorta; LV, left ventricle. Scale bar represents 20 µm.

Notch1 is expressed in the developing cardiac outflow tract and aortic valve during embryogenesis [14]. We sought to determine if Notch1 expression is maintained in the adult aortic valve. Radioactive section in situ hybridization was performed and demonstrated expression of Notch1 mRNA and its downstream target genes, Hey1 and Hey2, in the aortic valve of adult mice (Figure 1). Expression was seen in the leaflet interstitium and valve endothelium.

### Loss of NOTCH1 Localized to Areas of Calcification in Human CAVD

**Figure 2:**
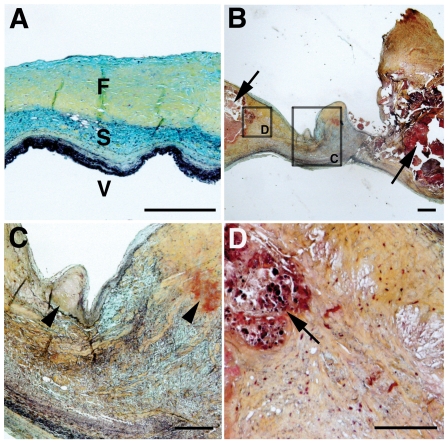
Calcific aortic valve disease demonstrates ECM disorganization and VIC disarray. Representative cross sections from control (A) and diseased (B) aortic valve cusps (3 control and diseased valves were examined). (C) and (D) are higher magnification images of boxed areas in (B). (A) Normal cusps have highly organized stratified ECM with fibrosa (F, yellow), spongiosa (S, blue), and ventricularis (V, black). (B, C) Diseased cusps have disorganized and dispersed ECM with increased collagens (yellow) and proteoglycans (blue) and decreased elastic fibers (black). Aortic valve cusp thickness is increased in diseased valves when compared to control valves (A, B). Calcification deposits are found in diseased valves (arrows, B) along with areas of early mineralization (arrowheads, C) and clusters of VICs that populate the margins of overt calcification (arrow, D). In general, the fibrosa appears expanded and calcification occurs in the arterial aspect of the cusp. The fibrosa is oriented upward in all panels, and the scale bars equal 500 microns in (A, B) and 250 microns in (C, D).

Histopathology with Movat's pentachrome stain demonstrated heterogeneous ECM disorganization in the diseased aortic valve when compared with controls (Figure 2). Specifically, normal ECM trilaminar stratification (Figure 2A) was lost as evidenced by the presence of increased and disorganized collagens and proteoglycans in all layers (Figure 2B). Cusp thickness was increased and variable in affected tissue. AVIC disarray was demonstrated in all aspects of the interstitium, and clusters of AVICs were seen in proximity to areas of early mineralization (Figure 2C) and overt calcification (Figure 2D). Evidence of calcification was confirmed by von Kossa and Alizarin red staining (data not shown). These findings demonstrate that CAVD is characterized by heterogenous disruption of ECM and AVIC organization, suggesting molecular mechanisms that regulate calcification processes may do so in a spatially restricted manner.

**Figure 3:**
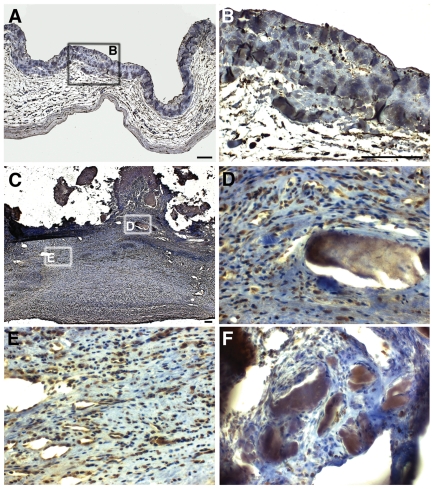
Loss of NOTCH1 expression in proximity to calcific nodules in human aortic valves. (A) Representative sections from control (A,B) and diseased (C-F) aortic valve cusps. (B) is high magnification image of boxed area in (A) and (D,E) are higher magnification of region in (C) while image in (F) shows another calcified aortic valve. Expression of Notch1 intracellular domain (NICD) is found in the thickened fibrosa of diseased aortic valve (C,E) as compared to the acellular fibrosa of control valves (A,B). However, there is significant loss of NICD expression in cells residing adjacent to calcific nodules (D,F). The fibrosa is oriented upward in all panels, and scale bars equal 100 microns (B,D,E,F are at same magnification). Brown signal represents NICD expression while nuclei are counterstained in blue.

To determine the expression of NOTCH1 in humans with CAVD, we obtained explanted valve tissue from adult patients at the time of aortic valve replacement. Aortic stenosis was the indication for surgery in all cases. We found a marked reduction in active NOTCH1, as measured by an antibody specific to the NOTCH1 intracellular domain (NICD), in regions localized to areas of calcification, which occurs in the fibrosa layer (Figure 3D and 3F) as compared to non-calcified fibrosa of diseased valves (Figure 3E). Of note, overall expression of NICD was increased in the fibrosa of diseased valves compared to the acellular fibrosa of controls, consistent with previous studies demonstrating increased cellularity, hyperproliferation, and increased valve cusp thickness in aortic valve disease (Figure 3A-C, 3E) [9], [23]. These findings demonstrate that CAVD is characterized by loss of NOTCH1 expression in areas of calcification along with ECM disorganization and VIC disarray.

### Notch Signaling Regulates Expression of Valve ECM Proteins

**Figure 4:**
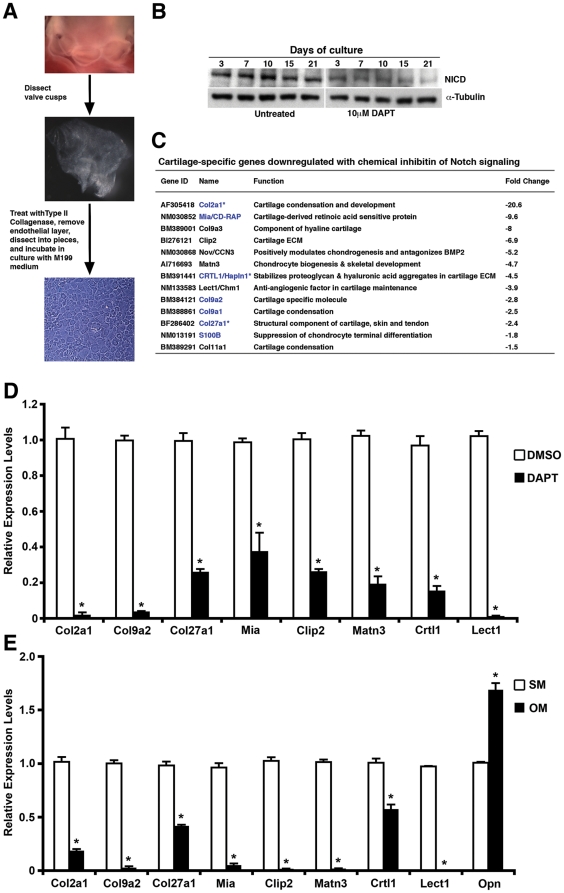
Identification of gene expression changes with inhibition of Notch signaling in rat AVICs. (A) Schematic showing the establishment of AVIC cultures from rat aortic valves. (B) Rat AVICs treated with DAPT demonstrate downregulation of Notch1 intracellular domain (NICD) when compared to untreated cells (DMSO only). Protein amounts were normalized to anti-α-tubulin. (C) RNA from control and DAPT-treated AVICs cultured for 10 days was extracted and analyzed for differential gene expression using Affymetrix Rat 230 2.0 GeneChip microarrays. Table showing fold downregulation of cartilage-specific genes with DAPT treatment of rat AVICs. In blue, direct transcriptional targets of Sox9; (*) genes with multiple hits. Decreased expression of cartilage-specific genes by qRT-PCR in AVICs with DAPT treatment (D) or when cultured in osteogenic media (OM) versus standard media (SM) (E). Expression of osteopontin (Opn) mRNA was upregulated in OM. *, p value<0.05. Experiments were performed in triplicate, and means and standard deviations are shown.

In order to identify the downstream mediators of Notch signaling in the early stages of aortic valve calcification, we applied an unbiased strategy using Affymetrix microarrays. Gene expression was performed with mRNA harvested at day 10 from rat AVICs treated with 10 µM of the γ-secretase inhibitor, DAPT diluted in DMSO, versus cells treated with DMSO alone, defined as control AVICs, using a previously reported culture protocol (Figure 4A) [7]. Downregulation of NICD expression in response to DAPT treatment was confirmed by immunoblotting prior to performing microarray studies (Figure 4B). Comparing Notch inhibited AVICs (DAPT-treated) versus control AVICs, there were 515 probe sets representing ∼470 genes that were differentially expressed (Table S1). A large portion of genes that were significantly altered are cartilage-specific genes that constitute the highly organized ECM in developing and mature aortic valve, including genes known to be involved in cartilage metabolism, bone mineralization and ossification (Figure 4C) [8]. No gene expression changes consistent with calcification were noted, likely secondary to the inability of rat AVICs to spontaneously calcify. The most profoundly downregulated gene, which was decreased over 20 fold, was Col2a1 (Type II collagen, alpha-1), an early marker of chondrocyte differentiation. Several other positive modulators of chondrogenesis or constituents of cartilage ECM were also downregulated including Type IX collagens, Mia/Cd-rap (melanoma inhibitory activity or cartilage-derived retinoic acid sensitive protein), Nov/Ccn3 (nephroblastoma overexpressed protein), Crtl1/Hapln1 (cartilage link protein/hyaluronic acid proteoglycan linking protein 1), Matn3 (Matrillin 3), Clip2 (cartilage intermediate layer protein 2) and Lect1/Chm-1 (leukocyte cell derived chemotaxin 1 or chondromodulin-1) (Figure 4C). Similar to NOTCH1, the expression of COL2A1 was downregulated in areas of calcification in diseased human aortic valves (Figure S1). The differential expression of several of these cartilage-specific ECM genes was further validated by quantitative real time RT-PCR (qRT-PCR) (Figure 4D), consistent with our findings of ECM dysregulation in diseased human aortic valves.

The expression of these genes was examined in rat AVICs grown in osteogenic media, which is required because AVICs of human and rodent origin develop cellular changes associated with calcification only by induction with organic phosphates [17]. As expected, osteogenic media resulted in increased expression of osteopontin, while the cartilage-specific ECM genes showed decreased transcript levels (Figure 4E), consistent with Notch inhibition studies in AVICs. Taken together, these findings suggest that loss of Notch signaling is associated with abnormal ECM gene expression in the aortic valve.

### Notch1 Regulates Sox9

**Figure 5:**
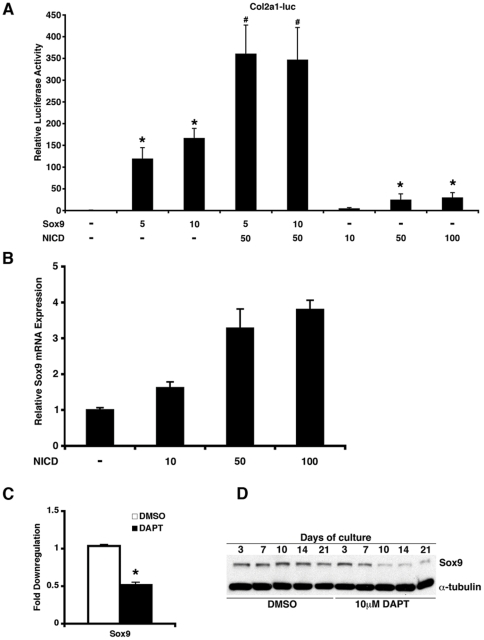
Notch1 regulates Sox9 expression in vitro. (A) Relative luciferase activity in COS7 cells transfected with Col2a1 luciferase reporter along with indicated amounts of Sox9 and Notch1 intracellular domain (NICD) expression plasmids. *, p value<0.05 comparing transfection of Sox9 or NICD with empty vector; #, p value<0.05 when comparing transfection of Sox9 and NICD with either Sox9 or NICD alone. (B) Transfection of increasing amounts of NICD expression plasmid upregulates Sox9 mRNA in COS7 cells as quantified by qRT-PCR. (C) Following treatment with DAPT, Sox9 mRNA was downregulated by qRT-PCR in rat AVICs after 10 days of culture. Mean and standard deviations are shown. *, p value<0.05. (D) Immunoblot analysis showing expression of Sox9 in rat AVICs over 21 days of culture treated with DAPT or DMSO (untreated). Protein amounts were normalized using α-tubulin antibodies. Experiments in A-C were performed in triplicate and means and standard deviations are shown. Transfected DNA amounts are in nanograms.

Investigation of Gene Ontology databases revealed that most of the cartilage-specific genes downregulated with DAPT treatment were also direct transcriptional targets of Sox9, a member of the Sox family of transcription factors that is expressed in chondroprogenitors and chondrocytes in addition to developing and mature valves (highlighted in blue in Figure 4C) [11], [12], [24], [25]. To determine if Notch1 could influence Sox9-dependent activation of cartilage-specific genes, we utilized the mouse Col2a1 luciferase reporter, which contains 4 tandem copies of the 48bp Col2a1 enhancer fragment (harboring Sox9 binding sites) upstream of the Col2a1 core promoter. Co-expression of constitutively active NICD with Sox9 resulted in synergistic activation of the luciferase reporter when compared to NICD or Sox9 alone (Figure 5A). Interestingly, we found that transfection of NICD alone resulted in low levels of reporter activation and this was dependent on the presence of the enhancer containing Sox9 binding sites (Figure 5A and Figure S2). Since the enhancer lacks any apparent RBP-Jk binding sites for direct activation by NICD and its coactivators, we postulated that increased reporter activation seen with NICD and Sox9 was partly the result of Sox9 upregulation by NICD. Accordingly, we found a dose-dependent upregulation of Sox9 mRNA in COS7 cells with overexpression of NICD by qRT-PCR (Figure 5B).

To determine if inhibition of Notch signaling affects expression of Sox9 in rat AVICs, we harvested mRNA after treatment with DAPT for 10 days. Quantitative RT-PCR demonstrated a two-fold downregulation of Sox9 mRNA in DAPT versus untreated cells (Figure 5C). This reduction in Sox9 was also observed at the protein level over 21 days of treatment with DAPT (Figure 5D). In addition to Sox9, transcript levels for Sox5 and Sox6, which are co-expressed with Sox9 and cooperate together to activate the Col2a1 enhancer in chondrogenic cells, were found to be decreased in AVICs with Notch inhibition (data not shown) [25]. Analysis of mouse Sox9 upstream regulatory region revealed a putative RBPjk binding site (Figure S3A). However, luciferase reporter assays in COS7 cells failed to demonstrate transactivation upon overexpression of NICD suggesting the Notch1 regulation of Sox9 was via an indirect mechanism (Figure S3B). These findings support a role of Notch1 in maintenance of Sox9 expression.

### Notch Signaling in the Calcification of Porcine AVICs

**Figure 6:**
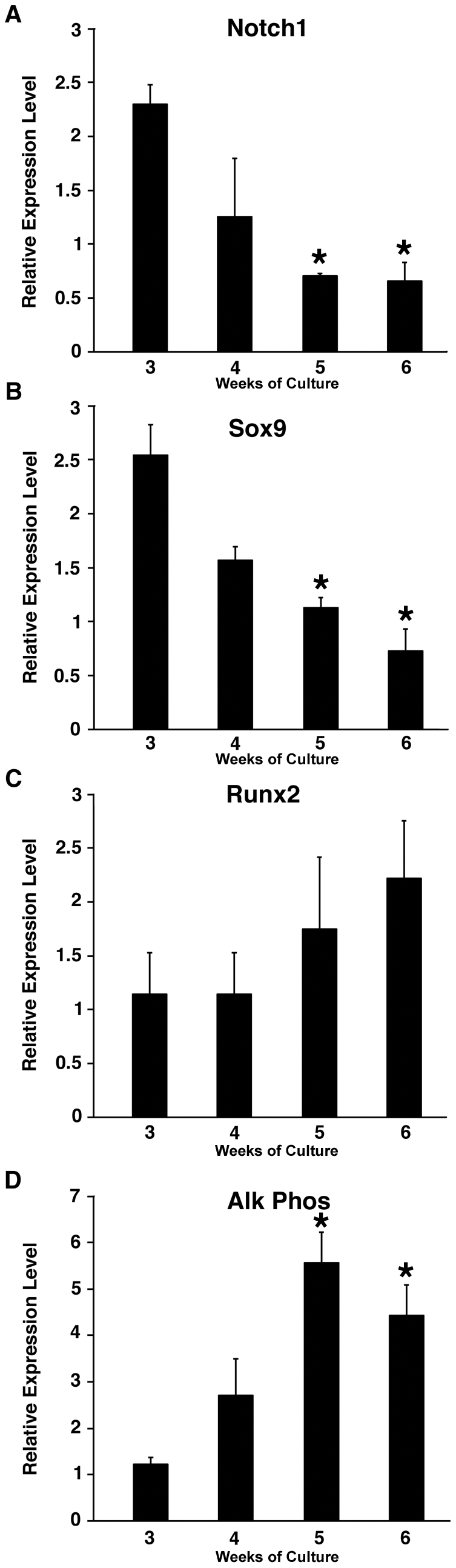
Loss of Notch1 and Sox9 expression is concomitant with increased expression of osteogenic markers in porcine AVIC culture. (**A**) Notch1 and (B) Sox9 mRNA levels significantly decrease over a 3-week time course as measured by qRT-PCR while increasing levels of (C) Runx2 and (D) alkaline phosphatase (ALP) mRNA were noted. Weeks of culture are noted on x-axis. Time course studies were performed triplicate and means and standard deviations are shown. *, p value < 0.05, when compared to week 3 levels.

In order to determine the role of Notch in valve calcification, we investigated whether members of the Notch signaling pathway could affect the calcification process in an established porcine AVIC calcification model system (Figure 6 and Figure S4A-C) [7]. Based on our human genetic studies identifying individuals with CAVD and NOTCH1 haploinsufficieny, we hypothesized that inhibition of the Notch signaling pathway would accelerate the process of calcification in this primary culture system. Interestingly, we observed that porcine AVICs, which are known to spontaneously calcify, display a gradual downregulation of Notch1 mRNA expression in concert with decreased expression of Sox9 even without DAPT treatment, which was concomitantly associated with increased expression of osteogenic markers, Runx2 and alkaline phosphatase (Figure 6). Treatment of AVICs with 10 µM DAPT, a chemical which blocks the release of the Notch intracellular domain (NICD), for 21 days resulted in elevated levels of osteopontin, a molecular marker associated with osteogenesis, and increased expression of osteonectin, which is found with in the early stages of calcification, when compared to untreated cells (Figure S4D and S4E). As has been previously reported, DAPT treatment resulted in an earlier rise in Runx2 and alkaline phosphatase mRNA levels and along with decreased levels of Sox9 mRNA (Figure S5) [26], [27]. To test if Notch signaling could inhibit valve calcification, the downstream targets Hey1 and Hey2 were overexpressed in porcine AVICs. We found that overexpression of Hey1 or Hey2 resulted in a decrease in osteopontin protein expression when compared to nucleofection of an empty vector or a GFP control vector (Figure S4G). Interestingly, we noted that Hey2 overexpression resulted in increased Sox9 mRNA expression but not Hey1 (Fig. S4H). Taken together, these loss and gain-of-function studies in a well-established cell culture model for valve calcification support the hypothesis that Notch signaling inhibits calcification of the aortic valve, consistent with the human genetic association of *NOTCH1* haploinsufficiency to CAVD.

### Overexpression of Sox9 decreases AVIC calcification that occurs with Notch inhibition

**Figure 7:**
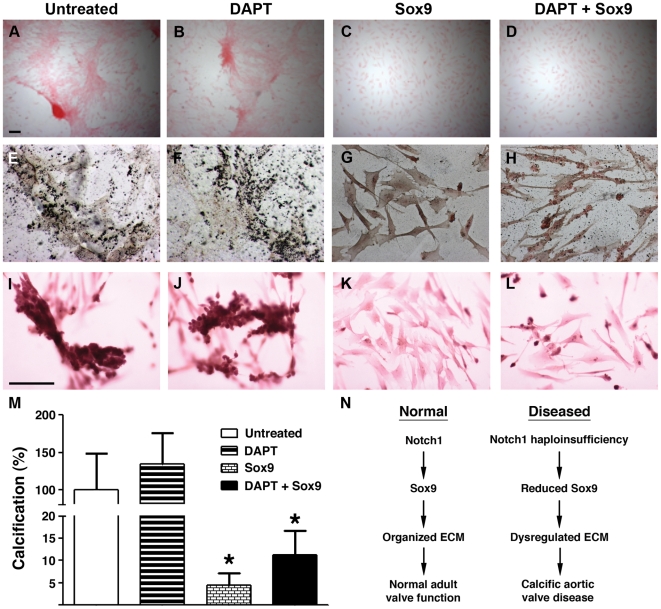
Overexpression of Sox9 rescues calcification that occurs with Notch inhibition. Porcine AVICs were cultured under four conditions: (A,E,I) untreated, (B,F,J) DAPT-treated, (C,G,K) Sox9 overexpression, and (D,H,L) DAPT-treatment following Sox9 overexpression. Bright field image of unstained cells (A-D), and cells stained with von Kossa (E-H) and Alizarin red (I-L) are shown. AVICs demonstrated calcification with calcific nodules in untreated and DAPT treated cells (A, B, E, F, I, J) but no calcification or nodules were found in Sox9 or DAPT/Sox9 treated cells (C, D, G, H, K, L). Three independent experiments were performed and representative images are shown. (M) Quantification of Alizarin red staining is shown. *, p value<0.05. (N) Schematic showing that Notch1-dependent activation of Sox9 is required for proper expression of valve ECM proteins that are essential for normal adult valve function. Conversely, loss of Notch1 or Sox9 results in dysregulated aortic valve ECM and CAVD.

To determine if Sox9 was functioning in a molecular pathway regulated by Notch signaling in aortic valve calcification, we investigated if overexpression of Sox9 could rescue the premature calcification that occurs with chemical inhibition of Notch signaling using the porcine AVIC calcification model system. Overexpression of Sox9 by nucleofection was able to severely attenuate calcification as examined by Von Kossa and Alizarin red staining in porcine AVICs treated with DAPT (Figure 7). Interestingly, porcine AVICs are known to spontaneously calcify in cell culture and the addition of Sox9 also prevented this unstimulated calcification. Transfection of Sox9 was confirmed by Western blot using antibody against Sox9 (data not shown). These studies show that loss of Sox9 is required for the accelerated calcification found with loss of Notch signaling in AVICs.

## Discussion

Mutations in *NOTCH1* have been linked to CAVD in humans, and Notch signaling is known to regulate multiple molecular pathways during embryonic and adult life including those critical for calcification [14], [28], [29]. Here, we demonstrate that Notch1 and its downstream mediators are expressed in the adult aortic valve and loss of Notch signaling is associated with areas of calcification in human aortic valves. Furthermore, we find that inhibition of Notch signaling downregulates the expression of Sox9, a key chondrogenic transcription factor that regulates the expression of ECM genes and is required for normal valve development, and promotes calcification in vitro. In addition, inhibition of Notch signaling in AVICs results in significant changes in the expression of cartilage-specific genes that comprise the valve ECM, which is altered in human CAVD. Lastly, we show that the addition of Sox9 can rescue the calcification that occurs in AVICs with Notch inhibition. These findings suggest a central role for Notch signaling in the prevention of valve calcification by regulation of Sox9.

Valvulogenesis is a complex process that begins during the early stages of cardiac development with the formation of endocardial cushions as a result of endothelial to mesenchymal transformation (EMT). Notch1 signaling is known to be critical for the cellularization of the endocardial cushions along with TGFβ, Wnt/β-catenin, and VEGF signaling pathways [30], [31]. During the later stages of embryonic development, mesenchymal cells diversify to give rise to distinct cell types and the highly organized ECM that constitutes the mature cardiac valve. The transcription factor, Sox9, guides the differentiation of mesenchymal cells into chondrocytes by activation of genes such as collagen types II, IX and XI, cartilage link protein and aggrecan that comprise the characteristic cartilage ECM. Murine studies have demonstrated a requirement for Sox9 during all stages of valvulogenesis as well as for maintenance of adult heart valves [11]–[13]. Germline inactivation of *Sox9* in mice results in embryonic lethality between embryonic day (E) 11.5 and 12.5 and hypoplastic endocardial cushions due to a failure of EMT, a phenotype similar to *Notch1-null* mice [11], [12], [30]. Inactivation of *Sox9* at later stages of development affects the expression of cartilage matrix-associated markers crucial for ECM maturation and diversification. Interestingly, Col2a1-*cre* mediated heterozygous loss of *Sox9* in adult mice leads to calcium deposition, a phenotype reminiscent of mice and humans harboring heterozygous null mutations in *NOTCH1* [13], [14], [26], [27]. More recent studies have demonstrated the role of retinoic acid in regulating Sox9 in the process of valve calcification [32]. Notch signaling has previously been shown to upregulate or downregulate Sox9 expression depending upon the cellular context [33], [34]. Consistent with this, diseased dysplastic valves demonstrate upregulation of Sox9 expression [35], similar to what we found with Notch1 (Figure 3). Expression of NICD in close proximity to areas of calcification is decreased, and more detailed examination of Sox9 expression in calcified human aortic valves is needed. Our studies in conjunction with the in vivo phenotypes found in mice and humans with loss of *Notch1* or *Sox9* suggest that Notch1 is necessary to maintain Sox9 expression in the process of valve calcification (Figure 7N). While our data do not support Sox9 as a direct target of Notch1, the Notch signal has been shown to be transduced through Bmp2, which is an important mediator of valve calcification [26]. Bmp2 has been shown to directly regulate Sox9 in chondrogenesis and additional studies are required to determine if a similar mechanism occurs during CAVD [36].

Mature valves exhibit a conserved trilaminar organization with each layer characterized by distinct ECM organization and mechanical properties [37]. On the aortic surface of the valve is the fibrosa layer, which is densely packed with various collagen fibrils that provide strength and stiffness, and imparts mechanical properties similar to bone. In contrast, the central spongiosa layer is enriched in proteolycans, enabling a more compressible matrix while maintaining structural integrity [8]. Several cartilage-specific proteins including cartilage link protein, Collagen type II and XI among several others are expressed in the spongiosa layer giving rise to molecular and structural properties similar to that of cartilage ECM. The ventricularis matrix layer composed mostly of elastic fibers is adjacent to the direction of blood flow and enables stretching and retraction of the leaflet during each cardiac cycle. This highly organized trilaminar ECM composition in turn provides the mechanical durability necessary for valve function. Supporting this notion, diseased valves invariably depict histopathological changes in ECM organization and VIC distribution [9], [23]. Disorganized ECM resulting from changes in proteoglycan, collagen and elastic fiber content have been shown to result in either stiff or floppy valves that lead to either stenosis or insufficiency, respectively [38]. In our studies, downregulation of Sox9 by Notch inhibition resulted in altered expression of multiple valve ECM proteins including Col2a1, Col9a1, Col11a2, Cd-rap and Crtl1 which are similar to the gene expression changes found in mice with targeted deletion of *Sox9* [12]. Additionally, enzymes involved in collagen maturation (P4ha3, collagen prolyl 4-hydroxylase alpha 3), proteoglycan synthesis (Has2, hyaluronan synthase 2) and collagen and elastin cross-linking (lysyl oxidases) also showed decreased expression along with other ECM components, such as elastin and fibronectin (Table S1), suggesting ECM abnormalities occur in all valve layers. Consistent with these observations, histopathological examination of aortic valve tissue from patients with CAVD shows ECM disorganization that results in a stiff stenotic valve, as seen in cases of *NOTCH1* haploinsufficiency.

Healthy, normal cardiac valves are avascular, and neovascularization of valves is a distinct histopathological characteristic of early valve disease and is associated with aortic valve calcification [39]. Consistent with this, we demonstrated upregulation of osteonectin, a molecular marker of neoangiogenisis and calcification with inhibition of Notch signaling (Figure S3F). In addition, an antiangiogenic factor, Lect1 (also known as Chondromodulin-1), was significantly downregulated in AVICs along with Sox9 in response to inhibition of Notch signaling and growth in osteogenic media (Figure 4C-E). Lect1 is known to be regulated by Sox9 during chondrogenesis and has a demonstrated role in CAVD as *Lect1*-null mice have increased neoangiogenesis, leaflet thickening and calcium deposition of valves with aging [40], [41]. Moreover, severe downregulation of Lect1 is observed in diseased cardiac valves [41]. Notch signaling has a well-documented function in guided vascular patterning and therefore, may also have a role in valvular disease by regulating neoangiogenesis [42].

Heterozygous mutations in *NOTCH1* have been linked to congenital malformations of the aortic valve along with adult-onset calcification [14], [43], [44], [45]. Our findings suggest that aortic valve ECM disorganization contributes to late-onset valve calcification. Consistent with this, bicuspid aortic valve, the most common cardiac malformation that often progresses to aortic stenosis and calcification has been shown to have disruption of the valvar ECM [11]. Ultimately, further elucidation of the Notch1-Sox9 molecular pathway and its role in the maintenance of the ECM will lead to an improved mechanistic understanding of aortic valve calcification and development of novel therapeutic strategies for CAVD.

## Supporting Information

Figure S1 **Loss of COL2A1 expression in proximity to calcific nodules in human aortic valves.** (A) Representative sections from control (A) and diseased (B-D) aortic valve cusps. (C, D) are high magnification images of boxed area in (B). Expression of the alpha-1 chain of type II collagen, (COL2A1) is found in the thickened fibrosa of diseased aortic valve (B) as compared to the acellular fibrosa of control valves (A). However, there is significant loss of COL2A1 expression in cells residing adjacent to calcific nodules (C) as compared to other regions lacking nodules (D). Scale bars equal 100 microns Brown signal represents COL2A1 expression while nuclei are counterstained in blue.
(JPG)

Figure S2 **Notch1 activation of Col2a1 luciferase reporter requires the presence of the enhancer containing Sox9 binding sites.** (A) Relative luciferase activity in COS7 cells transfected with luciferase reporter lacking enhancer fragment that contains Sox9 binding sites (Col2a1-lucΔEnh). Indicated amounts of Sox9 and Notch1 intracellular domain (NICD) expression plasmids used for transient transfection are shown.
(JPG)

Figure S3 **Notch1 does not activate Sox9 upstream regulatory sequences in vitro.** (A) Schematic of mouse Sox9 promoter region and luciferase reporter constructs generated. Mouse Sox9 core promoter (-272 to +1 bp) and 3 fragments containing the upstream sequence were cloned in pGL3basic luciferase reporter. *, putative RBPjk binding site; tss, transcription start site. (B) Relative luciferase levels of various constructs in COS7 cells with or without co-transfected NICD. All luciferase values were normalized with respect to core promoter activity. CP, core promoter.
(JPG)

Figure S4 **Porcine aortic valve interstitial cells (AVICs) spontaneously calcify and Notch signaling alters expression of osteogenic markers in AVICs.** (A) AVIC culture established from aortic valve leaflets dissected from 3 week old piglets. Cultured AVICs, a phenotypically diverse population of cells comprised of myofibroblasts, fibroblasts, and smooth muscles cells, transdifferentiate into osteoblast-like cells and undergo spontaneous calcification by forming calcified nodules (arrowheads) shown in low (B) and high (C) magnification. (D) Myofibroblast and osteoblast-specific cell markers in porcine AVICs harvested following 3, 10, and 21 days of culture. Myofibroblast markers vimentin and alpha-smooth muscle actin (α-SMA) were detectable early in culture. Increasing expression of osteoblast markers, osteopontin and the transcriptional regulator, Runx2, was noted after increasing days in culture. Protein amounts are normalized using GAPDH. (E) Earlier induction of osteopontin protein following Notch inhibition with γ-secretase inhibitor in AVICs when compared to untreated cells. Analysis of total cell lysate at days 3, 6, 10 and 21 by immunoblotting with anti-osteopontin antibodies. (F) Immunoblot analysis demonstrates increased osteonectin expression with treatment of AVICs with γ-secretase inhibitor (DAPT) compared to untreated cells. Days 3, 10, 15 and 21 are shown. (G) Decreased osteopontin protein by immunoblot with overexpression of Hey1 and Hey2 in AVICs after 10 days of culture when compared to nucleofections with no DNA, empty vector (pcDNA) and pmaxGFP. Protein amounts were normalized using GAPDH. (H) Increased Sox9 mRNA levels are found with overexpression of Hey2 but not Hey1 in pAVICs as quantified by qRT-PCR. Experiments were performed in triplicate and means and standard deviations are shown.
(JPG)

Figure S5 **Acceleration of calcification with Notch inhibition in porcine aortic valve interstitial cell culture system.** (A,B) Representative qRT-PCR showing higher levels of Runx2 and alkaline phosphatase (ALP) mRNA in DAPT-treated cells at weeks 3 and 4 as compared to control cells treated with DMSO. (C) Downregulation of Sox9 mRNA was also found with DAPT-treatment at 3 and 4 weeks of culture. Interestingly, Sox9 expression decreased over the 4-week time course as the cells calcified. Time course studies were performed twice and representative experiment is shown. qPCR studies were performed in duplicate and average is shown and expression levels are normalized to week 1 levels.
(JPG)

Table S1 **Gene expression changes with inhibition of Notch signaling in rat AVICs identified by Affymetrix microarray.**
(PDF)
