# Supplementary figures and images for: Inhibitory Role of Notch1 in Calcific Aortic Valve Disease
Source: PLoS One. 2011 Nov 16;6(11):e27743. doi: 10.1371/journal.pone.0027743 (PMC3218038; doi:10.1371/journal.pone.0027743)

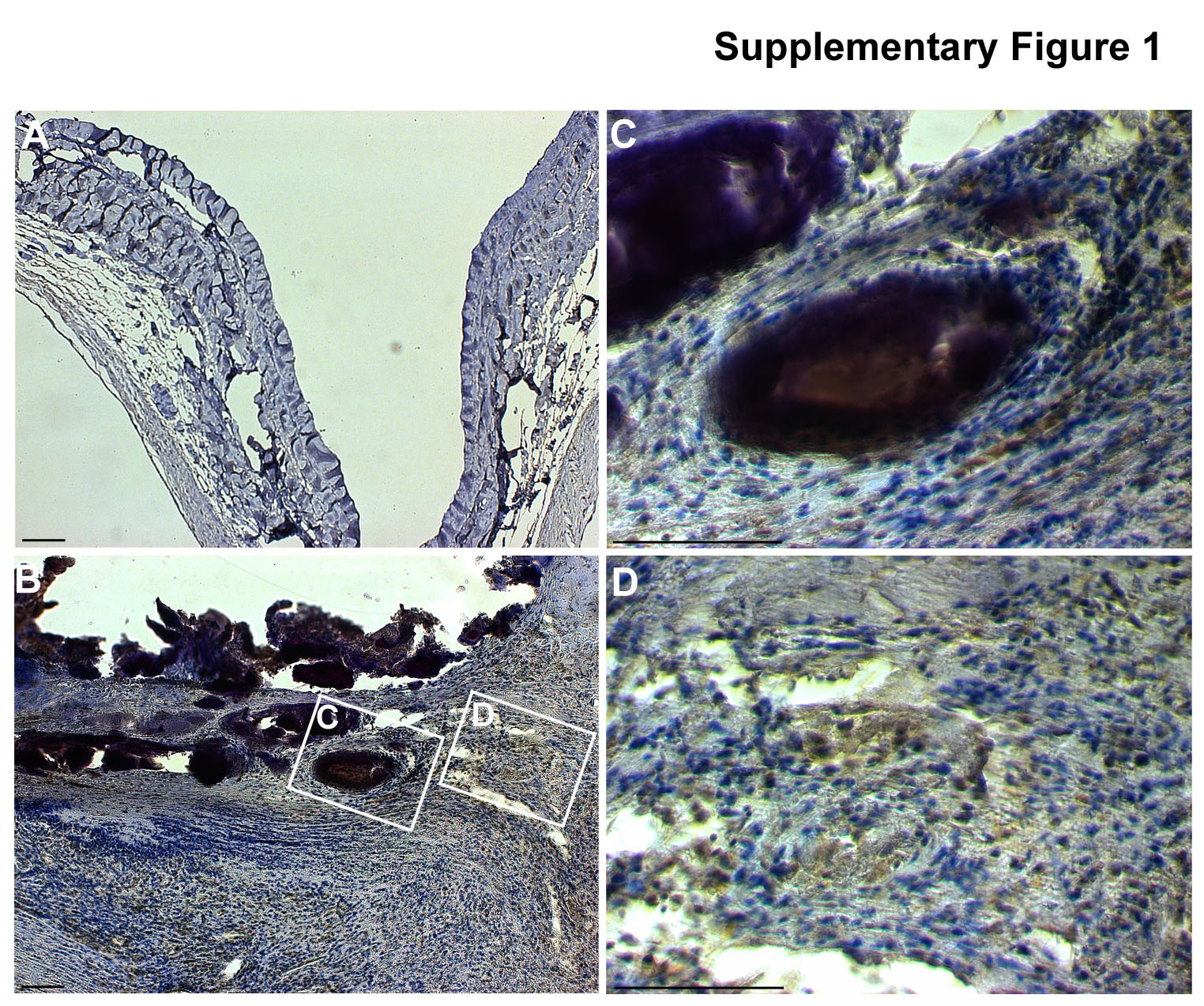

Supplement: Figure S1 — Loss of COL2A1 expression in proximity to calcific nodules in human aortic valves. (A) Representative sections from control (A) and diseased (B-D) aortic valve cusps. (C, D) are high magnification images of boxed area in (B). Expression of the alpha-1 chain of type II collagen, (COL2A1) is found in the thickened fibrosa of diseased aortic valve (B) as compared to the acellular fibrosa of control valves (A). However, there is significant loss of COL2A1 expression in cells residing adjacent to calcific nodules (C) as compared to other regions lacking nodules (D). Scale bars equal 100 microns Brown signal represents COL2A1 expression while nuclei are counterstained in blue. (JPG) [file pone.0027743.s001.jpg]

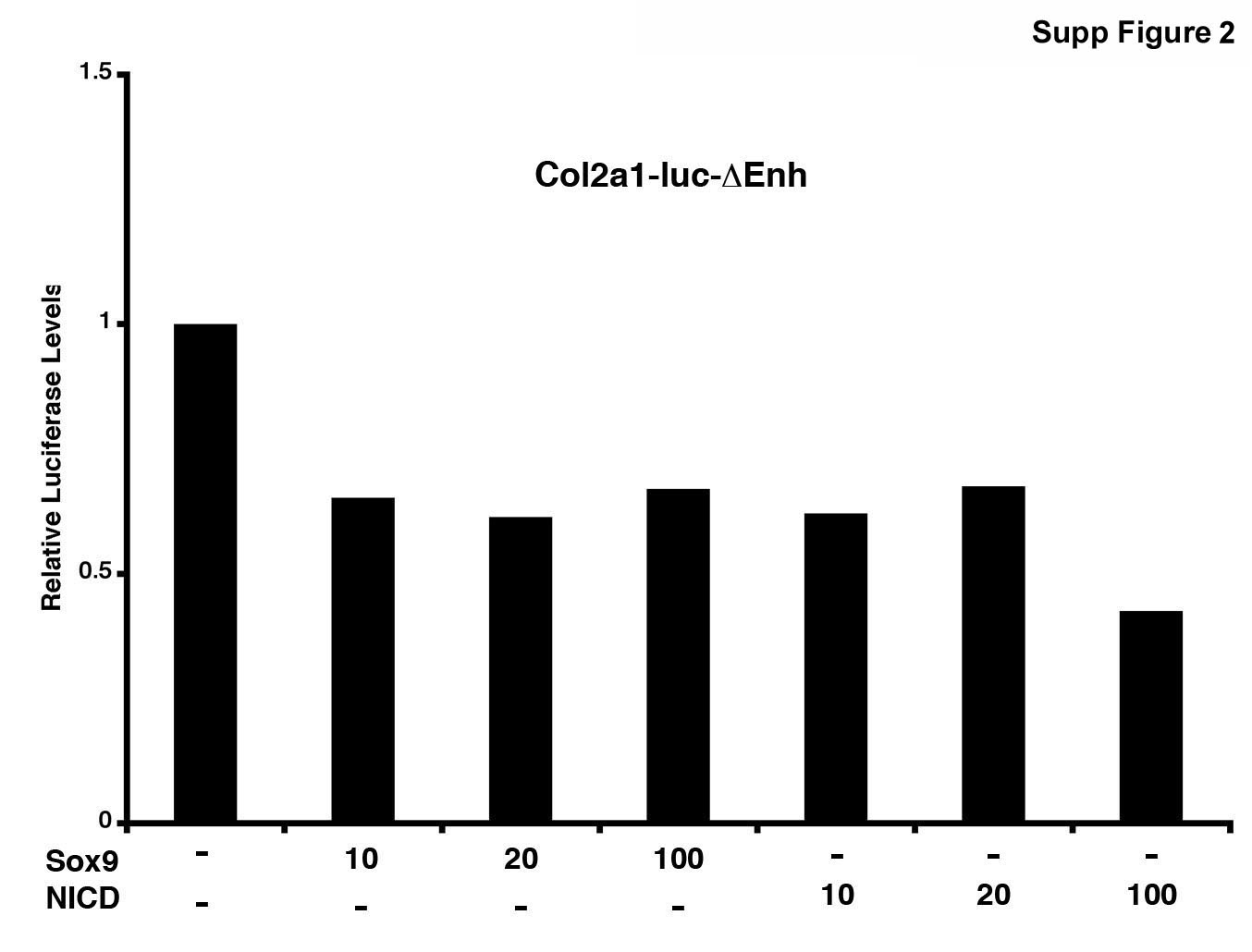

Supplement: Figure S2 — Notch1 activation of Col2a1 luciferase reporter requires the presence of the enhancer containing Sox9 binding sites. (A) Relative luciferase activity in COS7 cells transfected with luciferase reporter lacking enhancer fragment that contains Sox9 binding sites (Col2a1-lucΔEnh). Indicated amounts of Sox9 and Notch1 intracellular domain (NICD) expression plasmids used for transient transfection are shown. (JPG) [file pone.0027743.s002.jpg]

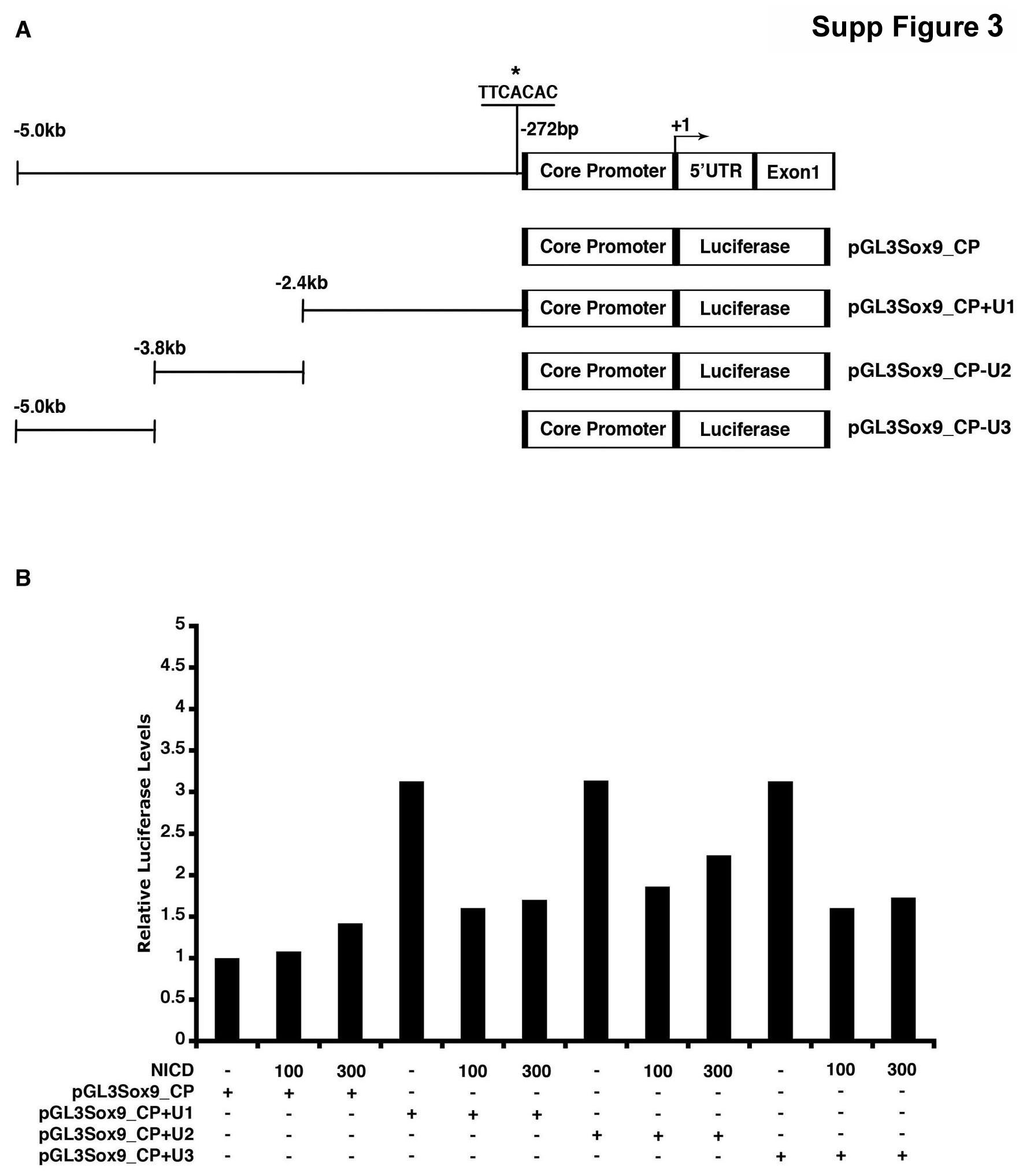

Supplement: Figure S3 — Notch1 does not activate Sox9 upstream regulatory sequences in vitro. (A) Schematic of mouse Sox9 promoter region and luciferase reporter constructs generated. Mouse Sox9 core promoter (-272 to +1 bp) and 3 fragments containing the upstream sequence were cloned in pGL3basic luciferase reporter. *, putative RBPjk binding site; tss, transcription start site. (B) Relative luciferase levels of various constructs in COS7 cells with or without co-transfected NICD. All luciferase values were normalized with respect to core promoter activity. CP, core promoter. (JPG) [file pone.0027743.s003.jpg]

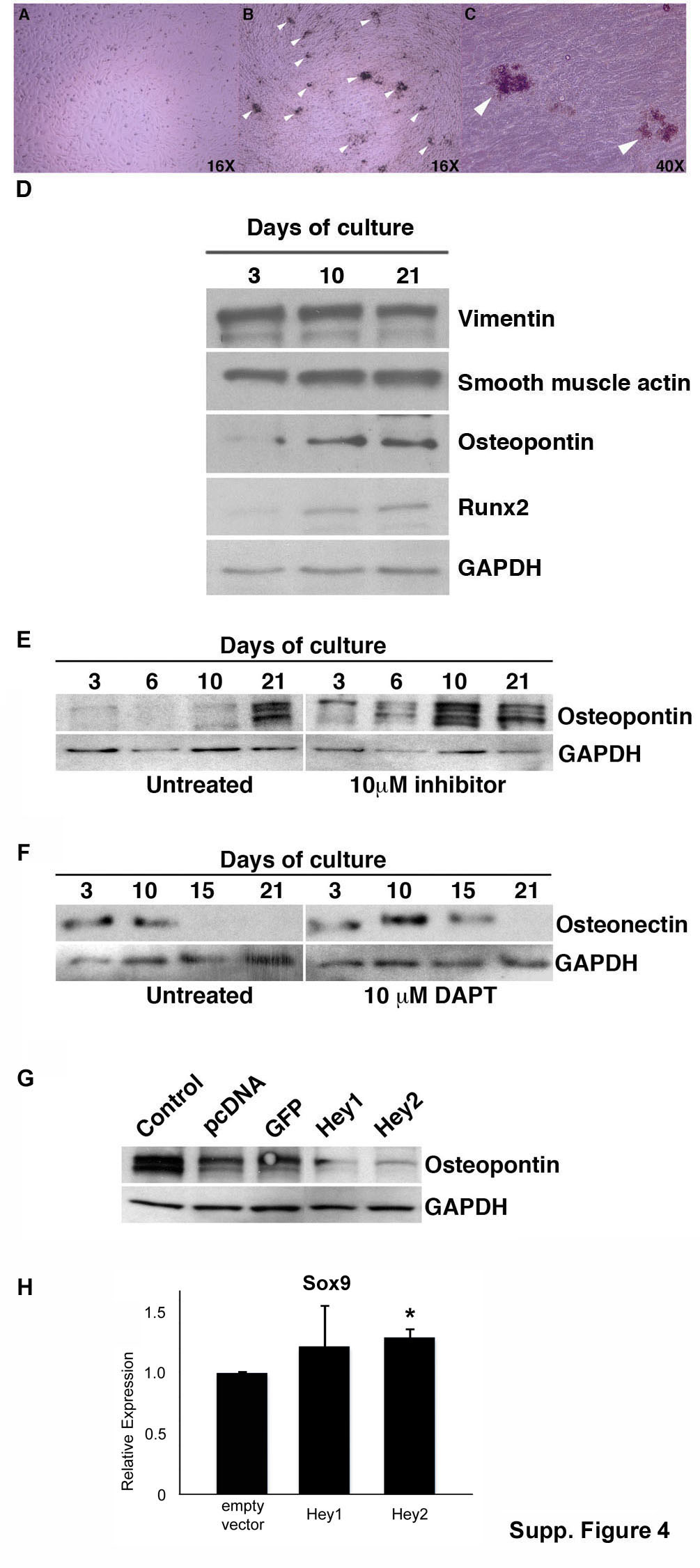

Supplement: Figure S4 — Porcine aortic valve interstitial cells (AVICs) spontaneously calcify and Notch signaling alters expression of osteogenic markers in AVICs. (A) AVIC culture established from aortic valve leaflets dissected from 3 week old piglets. Cultured AVICs, a phenotypically diverse population of cells comprised of myofibroblasts, fibroblasts, and smooth muscles cells, transdifferentiate into osteoblast-like cells and undergo spontaneous calcification by forming calcified nodules (arrowheads) shown in low (B) and high (C) magnification. (D) Myofibroblast and osteoblast-specific cell markers in porcine AVICs harvested following 3, 10, and 21 days of culture. Myofibroblast markers vimentin and alpha-smooth muscle actin (α-SMA) were detectable early in culture. Increasing expression of osteoblast markers, osteopontin and the transcriptional regulator, Runx2, was noted after increasing days in culture. Protein amounts are normalized using GAPDH. (E) Earlier induction of osteopontin protein following Notch inhibition with γ-secretase inhibitor in AVICs when compared to untreated cells. Analysis of total cell lysate at days 3, 6, 10 and 21 by immunoblotting with anti-osteopontin antibodies. (F) Immunoblot analysis demonstrates increased osteonectin expression with treatment of AVICs with γ-secretase inhibitor (DAPT) compared to untreated cells. Days 3, 10, 15 and 21 are shown. (G) Decreased osteopontin protein by immunoblot with overexpression of Hey1 and Hey2 in AVICs after 10 days of culture when compared to nucleofections with no DNA, empty vector (pcDNA) and pmaxGFP. Protein amounts were normalized using GAPDH. (H) Increased Sox9 mRNA levels are found with overexpression of Hey2 but not Hey1 in pAVICs as quantified by qRT-PCR. Experiments were performed in triplicate and means and standard deviations are shown. (JPG) [file pone.0027743.s004.jpg]

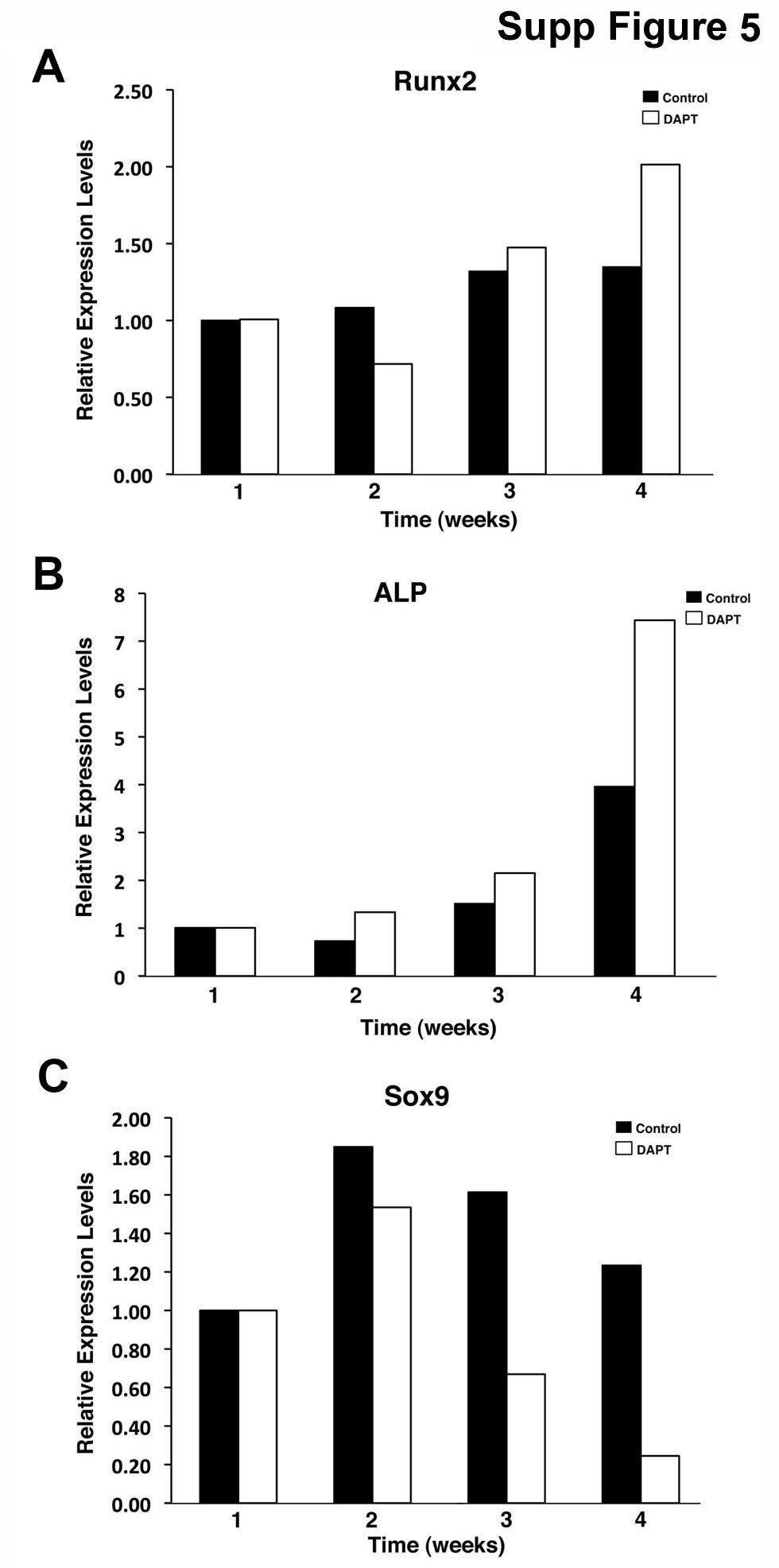

Supplement: Figure S5 — Acceleration of calcification with Notch inhibition in porcine aortic valve interstitial cell culture system. (A,B) Representative qRT-PCR showing higher levels of Runx2 and alkaline phosphatase (ALP) mRNA in DAPT-treated cells at weeks 3 and 4 as compared to control cells treated with DMSO. (C) Downregulation of Sox9 mRNA was also found with DAPT-treatment at 3 and 4 weeks of culture. Interestingly, Sox9 expression decreased over the 4-week time course as the cells calcified. Time course studies were performed twice and representative experiment is shown. qPCR studies were performed in duplicate and average is shown and expression levels are normalized to week 1 levels. (JPG) [file pone.0027743.s005.jpg]
