# Supplementary material for: Inhibitory Role of Notch1 in Calcific Aortic Valve Disease
Source: PLoS One. 2011 Nov 16;6(11):e27743. doi: 10.1371/journal.pone.0027743 (PMC3218038; doi:10.1371/journal.pone.0027743)
Supplement: Table S1 — Gene expression changes with inhibition of Notch signaling in rat AVICs identified by Affymetrix microarray. (PDF) [file pone.0027743.s006.pdf]

Supplementary Table. Gene expression changes with inhibition of Notch signaling in rat AVICs identified by Affymetrix microarray.

Gene ID	Name	Fold change	Gene ID	Name	Fold change
AY007690	Aard	-1.9	BI276121	Cilp2_predicted	-6.9
NM_013040	Abcc9	2.1	AA957183	Cit	-1.7
BI282702	Acta2	-2.6	AI705040	Cklfsf5_predicted	-5.4
NM_134329	Adh7	2.0	AI576758	Clec11a	-2.0
NM_012715	Adm	1.8	AF314657	Clu	3.0
AI175416	Agpat5_predicted	-1.6	AF314657	Clu	2.0
AW522526	Akr1cl1_predicted	3.5	BM389291	Col11a1	-1.5
NM_022407	Aldh1a1	-2.9	AA800298	Col15a1	1.8
NM_053896	Aldh1a2	-2.0	AW524253	Col23a1	3
NM_031972	Aldh3a1	2.6	BF392901	Col27a1	-2.2
NM_031544	Ampd3	2.0	BF286402	Col27a1	-2.4
L81174	Ankrd1	-2.5	AF305418	Col2a1	-20.6
NM_013220	Ankrd1	-2.3	AF305418	Col2a1	-7.4
NM_019363	Aox1	2.4	AI176126	Col6a3_predicted	1.7
NM_017123	Areg	1.9	BM388861	Col9a1	-2.5
BG377201	Arhgap24	-2.1	BM384121	Col9a2_predicted	-2.8
BI275896	Asam	-3.2	BM389001	Col9a3_predicted	-8
BI303342	Asam	-3.2	NM_012812	Cox6a2	2.4
AF329099	Asrgl1	-1.7	AF109674	Crispld2	-1.9
M14137	Atp1b1	-2.7	AA925924	Crlf1_predicted	-2.0
AI232036	Atp1b1	-2.3	AA866388	Crlf1_predicted	-2.0
NM_053019	Avpr1a	1.9	BE114076	Cttnbp2	-1.8
BF408665	Bace2	-1.8	NM_030845	Cxcl1	2.0
BI279562	Baiap2	1.8	AI171777	Cxcl12	3.5
NM_017178	Bmp2	3	AF189724	Cxcl12	4.5
NM_012827	Bmp4	-2	BF283398	Cxcl12	5.1
BI293047	Bmper_predicted	-2.6	X00469	Cyp1a1	2.4
BI292425	C1r	2.0	BF397093	Cyp26b1	-2.8
D88250	C1s	2.5	AA818353	Dapk1_predicted	2.2
NM_016994	C3	1.9	BM390253	Dcn	3.2
BI285347	C4a	3.9	NM_022297	Ddah1	-2.8
AB030829	Ca3	-3	NM_080399	Ddit4l	1.9
NM_019292	Ca3	-2.6	BI291018	Dok1	-1.8
NM_031808	Capn6	-1.9	AI138048	Dscr1l1	3.5
BM391835	Car9_predicted	-3.4	BI274408	Dscr1l1	4.5
BM387008	Casp3	1.9	AI231350	Dusp6	2.0
U84410	Casp3	1.9	NM_019234	Dync1i1	-2
AW915173	Cav	-1.6	NM_012947	Eef2k	1.7
BE098003	Ccbe1_predicted	1.7	BF284634	Efemp1	5.6
NM_031518	Cd200	-1.9	NM_022955	Egfl3	-1.9
BF417638	Cdca3	-1.7	AA957585	Ehd3	-1.9
BF402765	Cdh10	-3.3	BI274506	Eln	-3.5
BI296340	Cdh11	1.7	J04035	Eln	-2.7
BE111632	Cdh11	2.7	AF214568	Enpep	3.4
U23056	Ceacam10	-1.8	NM_053535	Enpp1	-1.8
L46791	Ces3	2.0	NM_019370	Enpp3	3.2

Gene ID	Name	Fold change
BI281250	Cftr	-1.9
BF284899	Cidea_predicted	2.3
AI715477	Eps8l1_predicted	-2.7
NM_133397	Erg	-2.4
AA963276	Etv1_predicted	2.5
AI059914	Etv1_predicted	2.7
NM_019199	Fgf18	-2.4
BM390970	Fgfrl1	-2.1
AF323608	Fgl2	1.8
BG663284	Fgl2	3.0
AI716194	Fgl2	2.4
AI411941	Fndc1	-4.4
M69056	Fntb	-4.1
NM_012743	Foxa2	1.8
NM_053629	Fstl3	-2.0
AW144239	Fstl3	-1.9
AI230396	Fyn	1.6
BI287978	Gadd45b	-1.9
AI101388	GalNAc4S6ST	1.9
NM_019216	Gdf15	2.3
NM_017009	Gfap	-1.7
NM_021654	Gja4	-1.8
BI284411	Glud1	1.7
NM_013145	Gnai1	2.4
AI112577	Gng3	-2.1
NM_012774	Gpc3	2.8
NM_030831	Gpcr12	-1.9
BF394166	Gpm6a	1.9
NM_133298	Gpnmb	2.2
BI284296	Gpr126_predicted	-1.8
NM_057201	Gpr37	1.6
NM_031696	Gpr88	3.5
NM_031623	Grb14	-2.1
NM_019189	Hapln1	-3.9
BM391441	Hapln1	-4.5
NM_013153	Has2	-2.0
AW434961	Hfe2	1.8
NM_022605	Hpse	2.0
AF177430	Hs3st1	2.5
NM_017080	Hsd11b1	2.0
NM_031971	Hspa1a	-2
NM_021863	Hspa2	-2
BF410146	Hspa2	-2.1
BE116009	Id4	1.8
AA943034	Igsf4c_predicted	-2.9
BF391914	Il1rap	-1.7
NM_013037	Il1rl1	-6.6
NM_022392	Insig1	1.7

Gene ID	Name	Fold change
NM_053927	Epb4.1l3	-3.4
AB032828	Epb4.1l3	-3.4
BI290037	Jam2	-1.8
NM_012971	Kcna4	6.5
AI010839	Kcnma1	-7
AW142820	Kirrel3_predicted	-2.6
NM_012696	Kng1	1.7
BF556962	Lama2_predicted	2
BI274917	Lama5	3.5
NM_130741	Lcn2	2.0
NM_030854	Lect1	-3.9
BI275904	Lims2	-1.8
AI602501	Lmcd1_predicted	-1.7
AB062135	LOC619393	2.4
AW528719	LOC680712	-1.6
BM388427	LOC680866	-1.8
AW527186	LOC685243	1.7
BE103244	LOC685611	-1.5
AI029494	LOC685899	1.7
BG371944	LOC686128	-1.8
BF393607	LOC689147	3.4
BM383531	LOC689415	1.8
AA957929	LOC689931	-2.7
BM387458	LOC690073	-1.7
AI717543	LOC691307	1.8
BI274399	Loxl3_predicted	-2
NM_012598	Lpl	2.1
BM383632	Lrrc48	-2.1
AI711152	Lrrn6a	-1.9
BG375362	Ltbp4	-2.1
BM390763	Mall	-4.1
NM_053847	Map3k8	2.1
AI575071	Me2_predicted	1.8
NM_017149	Meox2	2.2
AI716211	MGC105649	1.8
BE111332	MGC105733	-1.6
BM391248	MGC108778	-3.1
AI408440	MGC108823	2.9
AI556075	MGC112790	-2
AI385260	MGC72614	-1.9
NM_134349	Mgst1	3.9
NM_030852	Mia1	-9.6
BG378791	Mlana_predicted	2.7
NM_012608	Mme	-2.2
NM_133523	Mmp3	-2.9
NM_031055	Mmp9	-1.9
AW524012	Mrap_predicted	-2.1
AI408286	Ms4a7_predicted	-3.8

Gene ID	Name	Fold change	Gene ID	Name	Fold change
AI229643	Jak2	1.7	AF411318	Mt1a	2.4
AA849471	Jam2	-1.6	M24327	Mt1a	2.0
BG374290	Mtap2	-2.1	D78610	Ptpre	1.9
BE113032	Mtss1_predicted	1.6	AF239157	Rasd1	3.2
BF408465	Myct1_predicted	-2.3	BG375198	Rasl11b	-2
AI578120	Myh14	-2.3	BI286015	Rassf4	-2.0
BI279044	Myl9_predicted	-2.5	AI227769	Rassf4	-1.8
NM_133583	Ndrg2	-2.0	BG375029	Rassf4	-1.9
AW252250	Nebl_predicted	-1.9	NM_012733	Rbp1	-3.8
BM392374	Nedd9	-1.7	AI011920	Rcsd1_predicted	-1.7
BF555968	Nedd9	-1.8	AI059694	RGD1305459_predict	-2.7
NM_017029	Nef3	-3.2	BF418649	RGD1305645_predict	-1.7
NM_031522	Neu1	-1.9	BG380767	RGD1305664	-1.8
BE111846	Neu1	-1.8	BM390497	RGD1306323_predict	-3.1
BM389302	Nid2	-3.5	BG380826	RGD1306327	-2.1
AA859752	Nog	-1.8	BI295501	RGD1306658	1.9
NM_030868	Nov	-5.2	BF389238	RGD1306938_predict	2.8
X78595	Npr3	-1.9	BF415061	RGD1307034_predict	-1.7
NM_031073	Ntf3	2.3	BF408325	RGD1307569_predict	2.2
AW521702	Odz3_predicted	-1.7	AA963863	RGD1307618_predict	-4.2
NM_133306	Oldlr1	-6.8	AA963833	RGD1307618_predict	-3.5
AI711403	P4ha3	-2.5	BM383011	RGD1308428_predict	-2.1
BI281735	Pak1	-1.7	AW523875	RGD1309051	-1.6
U23443	Pak1	-1.8	AA955213	RGD1309362	2.8
AI176360	Palmd	-2	BM385387	RGD1309879	-2.2
AI231808	Palmd	-2.3	BM385779	RGD1311307	-2.0
AW919178	Palmd	-2.2	BE113624	RGD1311589_predict	1.8
BM388717	Panx3	-1.6	BM384131	RGD1311939_predict	-1.6
BI285321	Papss2_predicted	-2.9	BF398271	RGD1359691	-2.2
AW917486	Papss2_predicted	-3.3	BG377391	RGD1359691	-2.1
M83745	Pcsk1	2.2	BF283408	RGD1559803_predict	3.1
AA858930	Pde4b	-1.8	BE117002	RGD1560967_predict	-1.9
AB052170	Pdgfd	2.1	AI179609	RGD1561062_predict	1.7
BM384311	Pdgfrl	-1.5	AW521222	RGD1562115_predict	-1.8
NM_017139	Penk1	1.9	BF557676	RGD1562115_predict	-1.8
BG371843	Pkp1_predicted	-2.2	BM390001	RGD1562284_predict	1.8
BF289229	Pla2r1_predicted	-1.8	AI408343	RGD1562552_predict	2.0
NM_012760	Plagl1	-2.7	AI172174	RGD1562829_predict	1.9
NM_053758	Plce1	-2.5	AI170076	RGD1562829_predict	4.4
BF547014	Plekha6_predicted	2.0	BE107414	RGD1563246_predict	1.7
AF081582	Plekhb1	-3.4	H33003	RGD1563319_predict	-1.8
BM389026	Postn_predicted	-2.3	BF291123	RGD1563437_predict	2.7
NM_013124	Pparg	1.7	AW251654	RGD1565408_predict	-1.7
M12492	Prkar2b	2.0	BF282814	Rgl1_predicted	2.4
J04488	Ptgds	-2.0	BF544481	Rgl1_predicted	2.2
AI411541	Ptger3	2.9	AI706777	Rin3_predicted	-2.1
AB048730	Ptges	-1.8	NM_053338	Rrad	3.4
NM_017043	Ptgs1	1.9	BI288816	Rragd_predicted	2.3

Gene ID	Name	Fold change	Gene ID	Name	Fold change
NM_017066	Ptn	2.7	AJ243338	RT1-Aw2	2.3
AI104546	Ptp4a3_predicted	-1.6	NM_013191	S100b	-1.8
NM_033499	Scrg1	-4.9	AI030552	-	7.3
NM_013026	Sdc1	-1.8	BF404786	-	2.3
AW527486	Sema6a_predicted	1.9	BF391308	-	2.5
BM387083	Sema6a_predicted	3.9	BE104424	-	-4.6
AA859389	Sema6a_predicted	1.9	AI112936	-	2.2
AA799627	Sepp1	1.8	AI639128	-	2.6
NM_021696	Serpinb2	2.6	AI408151	-	-2.7
AW915763	Serping1	3.5	BE115264	-	4.5
BG378841	Sgca_predicted	1.9	BI303340	-	-1.8
AA800626	Sh2d4a	-1.7	AI236047	-	2
X97445	Slc16a7	2.6	BI298010	-	-3.6
NM_031664	Slc28a2	1.9	BI274660	-	1.6
NM_017217	Slc7a3	-2	AA944827	-	2.7
AI101171	Slco3a1	1.9	AI716693	-	-4.7
AF239219	Slco3a1	1.6	AA859437	-	2.2
AW915529	Slfn2_predicted	1.6	BF548045	-	2.4
AI175880	Smoc1	-2.2	BI292651	-	2.7
BG373119	Smoc1	-2	AW522471	-	-2.3
BM389498	Smpdl3a	2.0	AI145933	-	-2.8
AI639523	Spna1	-1.8	AA866443	-	-1.9
M88469	Spon1	5.6	BI295963	-	-2.7
M83143	St6gal1	-2.1	BF406252	-	2.7
NM_031549	Tagln	-2.9	BF403703	-	1.8
BG670310	Tgfa	-2.1	BI297236	-	1.9
NM_012671	Tgfa	-2.0	AW530225	-	1.9
NM_013174	Tgfb3	-2.7	BI295051	-	-1.8
BG379319	Tgfbi	3.8	AA956294	-	1.6
AI145313	Thy1	-1.8	BF560932	-	2.1
AW526982	Tlr2	1.8	AI502114	-	2.6
NM_053785	Tm4sf4	1.7	U18772	-	1.6
BE108949	Tmem30b_predicted	-2.3	BE107482	-	-5.8
BI274101	Tmepai_predicted	-2	BI279030	-	-5.4
AF159103	Tnfaip6	-3.1	AA955251	-	-4.1
NM_012676	Tnnt2	1.7	AI412779	-	-3.9
NM_023970	Trpv4	-1.6	BE113148	-	-3.5
BI285665	Ttyh3_predicted	1.9	BF406608	-	-3.2
NM_017237	Uchl1	-2.3	BI282114	-	-3
NM_012889	Vcam1	2.6	AA945604	-	-2.9
BI289085	Vnn1	1.7	AA943983	-	-2.8
BI279661	Wfdc1	-5.7	AI145639	-	-2.8
NM_022631	Wnt5a	2.2	AW532634	-	-2.7
AF228917	Zdhhc2	-2.2	BM387112	-	-2.6
BG375315	-	-1.9	BF398465	-	-2.6
BF405113	-	2.9	BI289110	-	-2.6
BF419584	-	3	BM389611	-	-2.6
AI502944	-	2	BI274989	-	-2.5

Gene ID	Name	Fold change
BM389075	-	-1.9
AA963069	-	2
AI175861	-	-2.3
AW536022	-	-2.3
BI296359	-	-2.2
AI574783	-	-2.1
AI410144	-	-2.1
BF395964	-	1.8
AW535897	-	1.8
AI170453	-	1.8
AI317817	-	1.8
AA944483	-	1.9
AW526548	-	1.9
BM386821	-	1.9
BI276554	-	2
BM383349	-	2.1
AI412164	-	2.1
AI555526	-	2.1
AI013328	-	2.1
BE120904	-	2.1
BF386238	-	2.2
BE108648	-	2.2
BF289002	-	2.2
BE109587	-	2.2
AI170446	-	2.2
AI043817	-	2.2
AI103530	-	2.3
BF562337	-	2.3
BF397954	-	2.4
BF394755	-	2.5
AA892496	-	2.5
AI170507	-	2.6
AI169239	-	2.8
AI704285	-	2.9
BE120930	-	2.9
BF403853	-	2.9
AA900536	-	2.9
BE103235	-	3.2
BM387260	-	3.4
AA819397	-	5.2
BI287026	-	5.7
BE102340	-	6.3
AA851385	-	2.4
AI233530	-	-5.2
BE108519	-	2.2
AA925323	-	-1.7
AA850766	-	1.9
BF398612	-	1.7

Gene ID	Name	Fold change
BF386659	-	-2.4
BG669921	-	-2.4
BG664827	-	-2.5
AI171219	-	1.9
BI281950	-	1.9
AI408442	-	2.7
BF402392	-	-2.2
AA858791	-	-3.4
AA891922	-	-2.3
BF281701	-	1.9
BG671943	-	1.9
AI102401	-	3.4
BI303075	-	2.5
BG666928	-	1.6
AW524891	-	-3.9
BE102375	-	-1.9
BM385137	-	-2.0
AW524543	-	-2.3
AA957424	-	-2.0
BE111121	-	-1.8
AW918480	-	1.7
BF283694	-	-1.6
AW916067	-	-1.8
AI071649	-	-1.7
BE104952	-	2.0
BI295140	-	-1.8
BE097091	-	-1.7
AW533021	-	-1.7
BF401102	-	-1.8
BI295810	-	-2.7
AI111767	-	-1.7
AI044318	-	1.8
AI029975	-	2.6
AA849479	-	-1.7
BI278180	-	2.0
BF291041	-	2.0
BE103875	-	1.7
AA996869	-	-2.1
AI501087	-	-1.6
BG380261	-	-1.9
AW526343	-	2.5
BI294158	-	1.8
AA924444	-	-4.5
AA925441	-	1.8
AA875124	-	-2.2
BG371790	-	-2.8
AI169118	-	1.9
BF407452	-	-1.8

Gene ID	Name	Fold change
AA899303	-	-9.3
AW919386	-	1.9
BE115243	-	1.7
BE113113	-	-1.6
AI171103	-	2.3
BF282184	-	-2.1
AA923988	-	2.4
AA963158	-	2.4
BF546029	-	1.7
AI146026	-	-1.9
BM388652	-	-1.7
AW920883	-	-2.0
BF390608	-	2.1
BF405828	-	1.8
BF391800	-	1.7
BI301147	-	1.7
AI713223	-	-2.2
BF411788	-	2.0
BI290825	-	-1.8
AI501537	-	1.8
AA848916	-	1.6
BI279680	-	-2.1
AA944568	-	1.7
AI144892	-	-2.8
BE110033	-	3.7
BG667725	-	-3.1
AW528601	-	-2.9
BI283881	-	-3.2
BE113272	-	-2.2
BE329099	-	-1.6
AW526050	-	1.8
BE108638	-	1.8
BE111725	-	-1.6
AI709793	-	-1.7
AA946437	-	1.6
BI275815	-	1.8
AI101727	-	1.8
AI010312	-	1.9
BG670247	-	-1.9
